# Clonal Clusters and Virulence Factors of Group C and G *Streptococcus* Causing Severe Infections, Manitoba, Canada, 2012–2014

**DOI:** 10.3201/eid2307.161259

**Published:** 2017-07

**Authors:** Sylvain A. Lother, Walter Demczuk, Irene Martin, Michael Mulvey, Brenden Dufault, Philippe Lagacé-Wiens, Yoav Keynan

**Affiliations:** AuUniversity of Manitoba, Winnipeg, Manitoba, Canada (S. Lother, B. Dufault, P. Lagacé-Wiens, Y. Keynan);; National Microbiology Laboratory, Winnipeg (W. Demczuk, I. Martin, M. Mulvey);; Diagnostic Service Manitoba, Winnipeg (P. Lagacé-Wiens)

**Keywords:** Streptococcus dysgalactiae, group C *Streptococcus*, group G *Streptococcus*, invasive disease, bacteremia, virulence factor, genome sequencing, Canada, bacteria

## Abstract

The incidence of group C and G *Streptococcus* (GCGS) bacteremia, which is associated with severe disease and death, is increasing. We characterized clinical features, outcomes, and genetic determinants of GCGS bacteremia for 89 patients in Winnipeg, Manitoba, Canada, who had GCGS bacteremia during 2012–2014. Of the 89 patients, 51% had bacteremia from skin and soft tissue, 70% had severe disease features, and 20% died. Whole-genome sequencing analysis was performed on isolates derived from 89 blood samples and 33 respiratory sample controls: 5 closely related genetic lineages were identified as being more likely to cause invasive disease than non-clade isolates (83% vs. 57%, p = 0.002). Virulence factors *cbp*, *fbp*, *speG*, *sicG*, *gfbA*, and *bca* clustered clonally into these clades. A clonal distribution of virulence factors may account for severe and fatal cases of bacteremia caused by invasive GCGS.

## Abstract 2

These strains are more likely to cause invasive infection, which is an emerging public health concern as incidence and disease severity are on the rise.

Group C and G *Streptococcus* (GCGS) are quickly becoming a major public health concern as the incidence of invasive infection and severe disease is increasing (*1*–*6*). In Manitoba, Canada, the incidence of GCGS bacteremia continues to increase, whereas the incidence of other invasive β-hemolytic streptococcal infections remains constant (*1*), similar to trends observed in Finland, Denmark, and Israel (*3*–*5*,*7*). These invasive infections cause severe illness, and up to 25% of patients die (*2*,*3*,*7*–*9*), yet the factors contributing to disease severity and death remain unclear.

*Streptococcus dysgalactiae* subsp. *equisimilis* (SDSE) is responsible for most cases of GCGS infections in humans (*10*,*11*). Historically considered nonpathogenic commensal flora, SDSE is now implicated in skin and soft tissue infections, pharyngitis, bacteremia, endocarditis, sepsis, toxic shock, and other invasive infections (*3*,*5*,*9*,*12*–*14*) that extensively overlap with the clinical presentations of *S*. *pyogenes* (group A *Streptococcus* [GAS]) infections. Similar to *S*. *pyogenes*, SDSE form large β-hemolytic colonies on sheep blood agar with hyaluronic acid capsules but express Lancefield group C or G carbohydrate (*15*) and possess M protein, which is vital in inhibiting complement pathway activation and resisting phagocytic killing (*16*). SDSE is genetically closely related to *S*. *pyogenes*, sharing 61%–72% sequence homology (*11*,*17*). These pathogens can exchange genes through bacterial phages and other mechanisms (*11*).

Approximately 71 virulence factor genes from *S*. *pyogenes* have been identified in SDSE, including hemolysin, streptolysin, exotoxin, proteinase, adhesin, streptokinase, and hyaluronic acid genes (*11*,*18*). *S*. *pyogenes* and SDSE carry streptolysin O (*slo*), which is required for invasive human infection (*11*,*19*), and streptolysin S (*sagA*), which has been linked to necrotizing soft tissue infections (*20*). Furthermore, the superantigen alleles *speA*, *C*, *G*, *H*, *I*, *K*, *L*, *M*, *N*, *O*, and *P*, which have been identified in *S*. *pyogenes*, have infrequently been identified in SDSE, but *speJ* and *ssa* are unique to GAS, and *szeN*, *szeP*, and *szeF* are unique to GCGS (*21*). The only commonly reported superantigen of SDSE is *speG* (*11*,*22*,*23*). Other commonly found virulence factors in SDSE are *lmb*, *gapC*, *sagA*, *hylB*, *slo*, *scpA*, and *ska*, whereas the presence of *cbp*, *fbp*, and *sicG* is variable and found only in a minority of strains (*22*). A conclusive association between virulence profile and disease propensity or site of isolation has not been demonstrated (*18*,*22*,*24*).

The monitoring of emerging pathogens requires phenotypic and molecular-based typing methodologies. Multilocus sequence typing (MLST) can be useful in tracking short-chain transmission of infections, but application of whole-genome sequencing for comparative studies provides higher resolution through a genomic epidemiology approach to investigate strain relatedness and dynamics. To uncover factors that may contribute to increased GCGS pathogenesis, we describe the clinical features of 89 GCGS bloodstream infections and the distribution of sequence types (STs) and virulence factors by whole-genome sequencing of 122 invasive and noninvasive isolates. We conducted this study in accordance with the ethical principles at the University of Manitoba after obtaining approval from the Health Research Ethics Board and Research Impact Committee.

## Materials and Methods

Using the records of 2 large laboratories, we retrospectively identified GCGS bacteremia cases that occurred during January 2012–December 2014 in Winnipeg, Manitoba, Canada. We identified 89 bacteremic events (defined as >1 blood culture positive for GCGS during a single hospital admission) among a total of 84 patients. We reviewed charts to obtain patient characteristics and clinical parameters for each bacteremic event. During September–December 2014, within the same geographic location as the study cohort, community physicians collected control pharyngeal swab samples from outpatients with signs or symptoms of pharyngitis. The samples, which were obtained at the physicians’ discretion, were cultured for identification of pyogenic streptococci: 33 noninvasive GCGS isolates were detected. These GCGS isolates were recovered from patients with symptomatic pharyngitis, but their symptoms were not severe and not necessarily attributable to GCGS. Although these control isolates were not from asymptomatic volunteers, the clinical differences between invasive blood stream isolates and noninvasive respiratory isolates was sufficient to compare genetic differences.

### Disease Severity

We considered patients with >1 of the following to have severe GCGS disease: in-hospital death, admission to intensive care unit, need for vasopressor or ventilatory support, diagnosis of streptococcal toxic shock syndrome (STSS) or infectious endocarditis, or a high-risk Simple Clinical Score >8 or Rapid Emergency Medicine Score >10. We defined STSS according to guidelines of the Working Group on Severe Streptococcal Infections (*25*). We calculated Simple Clinical Scores and Rapid Emergency Medicine Scores primarily by using patient vital signs and other clinical features; high-risk scores are associated with a 9.0%–10.3% risk for death by 30 days after admission (*26*–*28*).

### Collection and Identification of Bacteria

At the discretion of the healthcare provider, patient blood samples were collected at symptom onset into BacT/Alert bottles (bioMérieux, Saint-Laurent, QC, Canada) according to institutional protocol and incubated using the BacT/Alert blood culture instrument (bioMérieux). Isolates were stored in frozen stocks in skim milk at −70°C and later retrieved by subculture for further analysis. 

A total of 92 GCGS isolates were recorded during the study period; 90 were retrieved, 2 were lost in storage, and 1 was identified as *S. equi* subsp. *zooepidemicus* by 16S rRNA sequence similarity and excluded from the study. We plated the 89 remaining isolates onto sheep blood agar (Oxoid, Nepean, ON, Canada) and aerobically incubated them for 24 h at 37°C in the presence of 5% CO_2_. We confirmed isolate identification by using MALDI-TOF (matrix-assisted laser desorption/ionization time-of-flight) mass spectrometry with the MALDI BioTyper system (Bruker, Boston, MA, USA) according to the manufacturer’s protocol. To confirm isolates with ambiguous MALDI-TOF mass spectrometry identifications, we used latex agglutination to Lancefield antigens C and G and the Vitek2 system (bioMérieux) for biochemical identification. All isolates were identified as *S. dysgalactiae*.

### Whole-Genome Sequencing

We extracted DNA from cultures, created multiplexed libraries, assembled reads, and performed core nucleotide variation phylogenetic analyses (Technical Appendix 1). In brief, we generated paired-end, 300-bp indexed reads on the Illumina MiSeq platform (Illumina, San Diego, CA, USA); the average yield was 1,015,107 reads/genome, and the average genomic coverage was 145×. Read quality was assessed by using FastQC version 0.11.4 (http://www.bioinformatics.babraham.ac.uk/projects/fastqc/), assembled with SPAdes version 3.6.2 (http://cab.spbu.ru/software/spades/), and annotated with Prokka version 1.11 (http://www.vicbioinformatics.com/software.prokka.shtml), yielding an average contig length of 39,313 bp and an average N50 contig length of 82,867 bp (*29*–*31*). The high-quality reads were then mapped to the publically available reference genome, *S. dysgalactiae* subsp. *equisimilis* AC-2713 (GenBank accession no. NC_019042.1), by using SMALT version 0.7.5 (http://www.sanger.ac.uk/science/tools/smalt-0). Single-nucleotide variations (SNVs) were called using FreeBayes version 0.9.20 (https://github.com/ekg/freebayes) and SAMtools mpileup (http://www.htslib.org/) (*32*). The percentage of bases in the core was 82.8%, and 21,746 sites were used to generate the phylogeny.

We constructed a maximum-likelihood phylogenetic tree of informative SNV positions by using PhyML version 3.0 (http://www.atgc-montpellier.fr/phyml/) (*33*) and visualized the tree by using FigTree version 1.4.1 (http://tree.bio.ed.ac.uk/software/figtree/) (*34*). We determined phylogenetic clades by cluster analysis on the full dataset of blood and respiratory isolates (n = 122) and on isolates from blood only (n = 89) by using ClusterPicker version 1.2.4 (http://hiv.bio.ed.ac.uk/software.html) with the following settings: initial and main support thresholds = 0.9, genetic distance threshold = 4.5, and the large cluster threshold = 10 (*34*). We submitted whole-genome sequencing read data to the NCBI Sequence Read Archive (https://www.ncbi.nlm.nih.gov/sra/) under BioProject accession number PRJNA325743.

### Molecular Typing

We used the whole-genome sequencing data for in silico determination of MLST STs; virulence factors (*lmb*, *gapC*, *cba*, *cbp*, *fbp*, *sagA*, *slo*, *hylB*, *spegg*, *sicG*, *fbsA*, *pavA*, *fnbA*, *fnbB*, *gfbA*, *scpA*, *scpB*, *bca*, *cylE*, *ska*, *skc* and *skg*) (*22*,*35*); and superantigens (*speA*, *speB*, *speC*, *speF*, *spegg*, *speH*, *speI*, *speJ*, *speL*, *mf*-*2*, *mf*-*3*, and *smeZ*) (*21*,*23*). We determined Lancefield serogroups from sequences annotated with Prokka and confirmed them by serologic testing using commercial latex antisera (SSI Diagnostica, Hillerød, Denmark). We submitted MLST allelic profiles to the *Streptococcus dysgalactiae* MLST database (https://pubmlst.org/sdysgalactiae/). We used allelic profiles to compute a goeBURST (global optimal eBurst; http://www.phyloviz.net/goeburst/) full minimum spanning tree using PHYLOViZ (http://www.phyloviz.net/) (*36*); groups were assigned by a single-locus variation from a founding ST. All strains were confirmed to belong to *S*. *dysgalactiae* subsp. *equisimilis* by BLASTn (*37*) alignment of 16S rRNA sequences to reference genomes of *S*. *dysgalactiae* subsp. *dysgalactiae* ATCC27957 and *S*. *dysgalactiae* subsp. *equisimilis* ATCC12394 (PubMed accession nos. NZ_CM001076.1 and NC_017567.1, respectively).

### Statistical Methods

We used descriptive statistics, χ^2^ test, Kruskal-Wallis test, and Fisher exact test to compare demographics between clusters of SDSE to determine whether they differed with respect to key risk factors. We used Fisher exact test to compare risk of death and other disease severity markers between ST clusters and clades. No observations were censored, so survival analysis techniques were not necessary.

## Results

### Patient Characteristics and Disease Severity

We investigated 89 GCGS bacteremic events in 84 patients in Winnipeg during 2012–2014. Most patients (63%) were male, and the mean age was 61 years (SD ± 18.4 years). Many patients had co-existing conditions, predominantly cardiovascular disease (47%) and diabetes mellitus (43%). The most common source of bacteremia was from skin and soft tissue infections (51%), and 37% of patients had primary bacteremia. Infectious endocarditis was confirmed or suspected in 7% of patients. No patients had necrotizing fasciitis or pharyngitis (Table 1).

**Table 1:** Demographic and other variables among patients with group C and G *Streptococcus* bacteremia causing severe infections, Winnipeg, Manitoba, Canada, 2012–2014*

Patient variable	Value
Demographic characteristic	
Median age, y ± SD	61 ± 18.4 (0–99)
Age groups, y	
<18	1/89 (1)
18–64	52/89 (58)
>65	36/89 (40)
Sex	
M	56/89 (63)
F	33/89 (37)
Medical history†	
Active alcohol abuse	12/88 (14)
Active malignancy	16/88 (18)
Active smoker	17/88 (19)
Asthma or COPD	12/88 (14)
Cardiovascular disease	41/88 (47)
Chronic kidney disease	25/88 (28)
Diabetes mellitus	38/88 (43)
Dialysis dependent	10/88 (11)
History of intravenous drug use	3/88 (3)
Immunosuppressive drug use	11/88 (13)
Total parental nutrition	3/88 (3)
No predisposing conditions	8/88 (9)
Clinical source of bacteremia‡	
Skin and soft tissue infection	43/84 (51)
Intraabdominal or gastrointestinal infection	3/84 (4)
Pharyngitis	0/84
Osteomyelitis and discitis	1/84 (1)
Meningitis	1/84 (1)
Septic arthritis	2/84 (2)
Infectious endocarditis	6/84 (7)
Primary bacteremia without source	31/84 (37)
Clinical characteristic§	
Temperature >38°C	48/83 (58)
Mean arterial pressure <80 mm Hg	50/82 (61)
Heart rate >90 beats/min	63/83 (76)
Glasgow Coma Scale <15	36/84 (43)

*Values are no. patients in category/total no. patients with data available (%) except as indicated. COPD, chronic obstructive pulmonary disease. †Data missing for 1 patient. ‡Data missing for 5 patients. Data missing or partially missing for 7 patients.

In 70% of the cases, bacteremia was associated with markers of severe disease, including admission to an intensive care unit (26%) and the need for vasopressor (19%) or ventilatory (17%) support. Seventeen percent of patients had a diagnosis of STSS, and 35%–61% of patients had high-risk disease severity scores. Twenty percent of patients with GCGS bacteremia died while in the hospital (Table 2).

**Table 2:** Death and markers of disease severity among patients with group C and G *Streptococcus* bacteremia causing severe infections, Winnipeg, Manitoba, Canada, 2012–2014*

Disease severity marker	Value
Death	18/89 (20)
Severe disease	62/89 (70)
Streptococcal toxic shock syndrome†	14/82 (17)
Rapid Emergency Medicine Score >10†	29/82 (35)
High risk Simple Clinical Score >8†	50/82 (61)
Vasopressor support required†	16/84 (19)
Ventilatory support required†	14/84 (17)
Admission to intensive care unit required†	22/84 (26)

*Values are no. patients in category/total no. patients with data available (%) except as indicated. †Data missing or partially missing for 5–7 patients. A Rapid Emergency Medicine Score >10 and Simple Clinical Score >8 is considered high risk and associated with a 9.0%–10.3% risk of death.

### SDSE Isolate Characteristics

SDSE isolates from blood represented 89 (73%) of 122 total isolates; 33 (37%) of the 89 isolates were from female patients and 56 (63%) were from male patients. These isolates were classified as Lancefield groups G (63%) and C (37%). Respiratory isolates represented 27% (33/122) of the isolates; information regarding the number from female and male patients was not available. These isolates also were classified as Lancefield groups G (52%) and C (48%).

### Core Single-Nucleotide Variation Phylogenetic Analysis

Phylogenetic analysis of all 122 isolates showed no association between infection type and patient sex, age, or disease severity (Technical Appendix 1 Figure). Compared with the heterogeneous nonclade isolates, those that clustered into clades A–E represented a higher proportion of blood isolates (25/45 [57%] vs. 64/77 [83%], respectively; p = 0.002). In addition, compared with the other clades combined, clade A was represented by significantly fewer blood isolates (36/38 [95%] vs. 28/39 [72%], respectively; p = 0.017). In silico molecular determinants (MLST, Lancefield serogroups, and virulence factors) were clustered in a clonal distribution (Technical Appendix 1 Figure). However, we found no significant associations when comparing blood and respiratory isolates.

**Figure 1:**
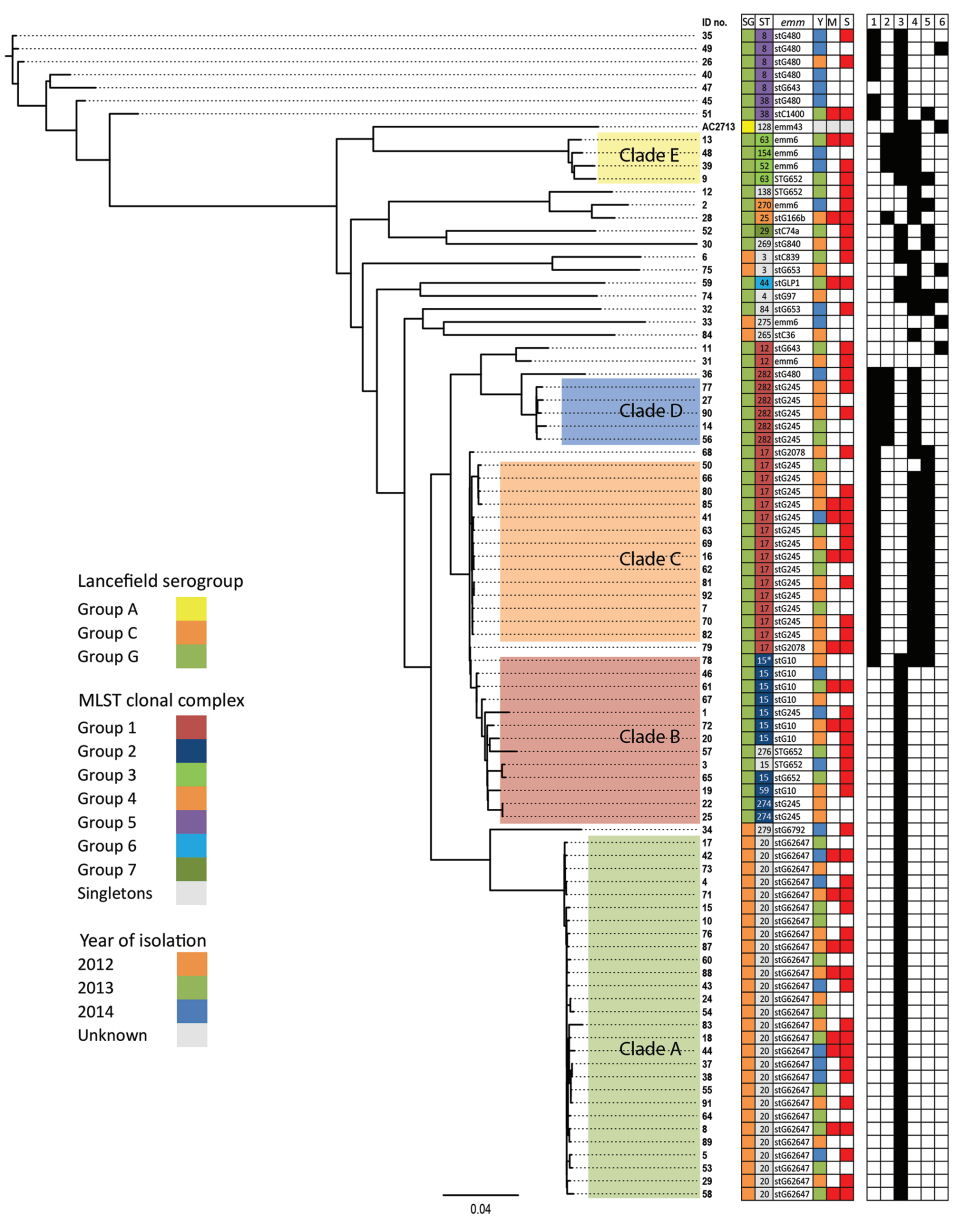
Maximum-likelihood whole-genome, core single-nucleotide variation (SNV) phylogenetic tree of 89 *Streptococcus dysgalactiae* subsp. *equisimilis* isolates from the blood of patients with group C and G *Streptococcus* causing severe infections, Winnipeg, Manitoba, Canada, 2012–2014. Multilocus sequence typing clonal complex relatedness groups were determined by using goeBURST (global optimal eBurst; http://www.phyloviz.net). In the mortality column, red and white squares indicate patient death and survival, respectively. In the severity column, red and white squares represent manifestation of severe and nonsevere disease, respectively. Black and white squares indicate the presence and absence of virulence factor genes, respectively. Scale bar indicates estimated evolutionary divergence between isolates, based on the average genetic distance between strains (estimated substitutions in sample/total high-quality SNVs). ICU, intensive care unit; IE, infectious endocarditis; MLST, multilocus sequence type; STSS, streptococcal toxic shock syndrome; SG, serogroup; ST, MLST; Y, year; M, mortality; S, severity; 1, *cbp*; 2, *fbp*; 3, *speG*; 4, *sicG*; 5, *gfbA*; 6, *bca*.

Cluster analysis of the 89 blood isolates yielded 5 clades, A–E (n = 64); the other 25 heterogeneous isolates were outside these lineages. Clade A isolates were Lancefield serogroup C, clades B–E were serogroup G, and the heterogeneous nonclade isolates were serogroups C (n = 5) and G (n = 20) (Figure 1). Isolate numbers 35, 49, 26, 40, 47, 45, and 51 were most genetically distant from the other blood isolates, averaging 3,897–3,987 SNVs. The greatest difference was 5,110 SNVs between isolate numbers 30 and 51 (Technical Appendix 2). Clade C was the most genetically homogenous, showing a maximum of 138 SNVs between isolates in the clade. Clade B was the most diverse, showing a maximum difference of 600 SNVs between isolates (online Technical Appendix Table 1).

### MLST

**Figure 2:**
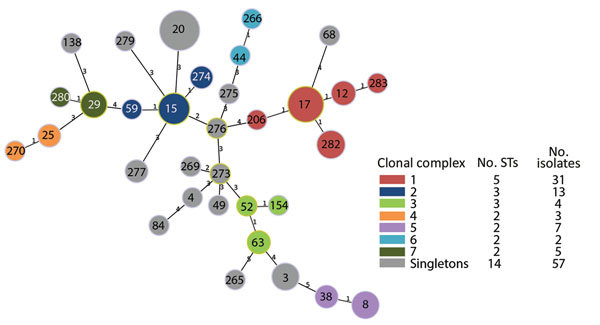
Minimum spanning tree representing the genetic relatedness of multilocus sequence types (MLSTs) of *Streptococcus dysgalactiae* subsp. *equisimilis* isolates from patients with group C and G *Streptococcus* causing severe infections, Winnipeg, Manitoba, Canada, 2012–2014. Genetic relatedness was determined by full goeBURST (global optimal eBurst; http://www.phyloviz.net) analysis using *Streptococcus dysgalactiae* MLST allelic profiles of 7 housekeeping genes. Numbers on nodes correspond to individual sequence types (STs) and colored nodes correspond to clonal cluster relatedness groups defined by a single-locus variation from a founding ST. Number labels on branches indicate the number of allelic variations between STs; branch lengths are not to scale.

STs for all 122 isolates generally correlated with specific phylogenetic clades and subclades (Figure 1; Technical Appendix 1 Figure). The most common STs were ST20 (n = 28), followed by ST17 (n = 16) and ST15 (n = 9) (Figure 2). Clade A (n = 28) consisted entirely of ST20 isolates belonging to a singleton MLST relatedness group. Clade B (n = 13) belonged to MLST clonal complex (CC) 2, in which ST15 (n = 9), ST69 (n = 1), and ST274 (n = 2) isolates grouped into subclades. An isolate with ST276 (a double-locus variant of ST15) also clustered into clade B. Clades C (n = 14) and D (n = 5) belonged to MLST CC1; clade C consisted of ST17 isolates, and clade D consisted of ST282 isolates. Clade E (n = 4) belonged to MLST CC3, in which ST63 (n = 2), ST52 (n = 1), and ST164 (n = 1) isolates grouped into subclades. Although SNV phylogenetic analysis showed that ST17 (clade C) and ST15 (clade B) isolates were closely related, large variations in MLST separated them into distinct clonal clusters (Figure 2). 

**Figure 3:**
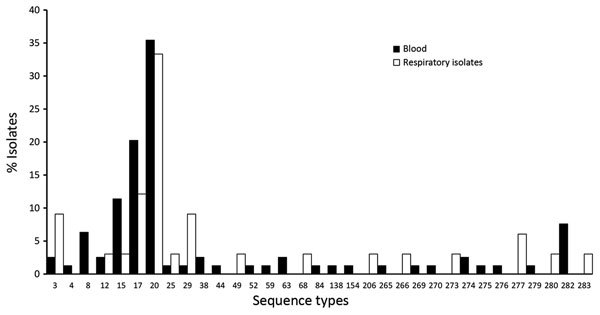
Prevalence of sequence types, as characterized by multilocus sequence typing, among blood and respiratory isolates of *Streptococcus dysgalactiae* subsp. *equisimilis* from patients with group C and G *Streptococcus* causing severe infections, Winnipeg, Manitoba, Canada, 2012–2014.

A total of 18 STs were unique to blood isolates: STs 4, 8, 38, 44, 52, 59, 63, 84, 138, 154, 265, 269, 270, 274, 275, 276, 279, and 282. A total of 8 STs were unique to respiratory isolates: STs 49, 68, 206, 266, 273, 277, 280, and 283 (Figure 3).

### Invasive Polymicrobial Infections

Polymicrobial bacteremia with organisms other than GCGS alone was present in 18% (16/89) of patients. In 4 patients with non-GCGS organisms plus GCGS isolates (i.e., isolate nos. 3 and 57, which clustered in clade B; and nonclade nos. 12 and 74), the non-GCGS organisms were believed to represent 1) contaminants at the time of sample collection or 2) the nonprimary pathogen. *Staphylococcus aureus* co-infection was seen in 6 patients. Four patients had GCGS isolates that clustered into clade C (nos. 41, 70, 82, 85), and the isolates were all associated with severe disease features (Technical Appendix 1 Table 2). Two of the 4 patients died.

### Distribution of Virulence Factors

All 122 isolates carried virulence factors *gapC*, *hylB*, *lmb*, *sagA*, *scpA*, *scpB*, *ska*, *skc*, *skg*, and *slo*; however, virulence factors *cba*, *cfb*, *cylE*, *fbsA*, *fnbA*, and *pavA* were universally absent. Other factors were variably present (Table 3). Factors *cbp*, *fbp*, *speG*, *sicG*, *gfbA*, and *bca* clustered clonally into the phylogeny (Figure 1). All clade A and B isolates contained only *speG*, with the exception of 1 clade B isolate that also contained *cbp*, *sicG*, and *gfbA*. Clade C consisted of isolates with *cbp*, *sicG*, and *gfbA*; clade D isolates had *cbp*, *fbp*, and *sicG*; and clade E isolates had *fbp*, *speG*, and *sicG*. The virulence factor *fbp* was present in clades D and E and in 1 nonclade isolate (no. 28). Virulence factor *bca* was found variably in 5 nonclade isolates (nos. 49, 75, 74, 33, and 11) and in the reference isolate, AC-2713, which also contained genes *speG* and *sicG*. No association was discovered between the presence of these virulence factors and disease severity.

**Table 3:** Distribution of virulence factor genes in blood and respiratory isolates of *Streptococcus dysgalactiae* subsp. *equisimilis* from patients with group C and G *Streptococcus* bacteremia causing severe infections, Winnipeg, Manitoba, Canada, 2012–2014

Gene	Gene product	No. isolates positive for virulence factor/no. tested (%)			Reference
		Total isolates	Blood isolate	Respiratory isolate	
Adhesins					
* gapC*	Glyceraldehyde 3-P dehydrogenase	122/122 (100)	89/89 (100)	33/33 (100)	(*22*)
* Lmb*	Laminin-binding surface protein	122/122 (100)	89/89 (100)	33/33 (100)	(*22*)
* fnbB*	Fibronectin-binding protein	120/122 (98.4)	89/89 (100)	31/33 (94)	(*35*)
* fnB*	Fibronectin-binding protein	120/122 (98.4)	89/89 (100)	31/33 (94)	(*35*)
* cbp*	Collagen-binding protein	34/122 (27.9)	29/89 (33)	5/33 (15)	(*22*)
* gfbA*	Fibronectin-binding protein	32/122 (26.2)	24/89 (30)	8/33 (24)	(*35*)
* fbp*	Fibronectin-binding protein	11/122 (9.0)	10/89 (11)	1/33 (3)	(*22*)
* fbsA*	Fibrinogen-binding protein	0/122	0/89	0/33	(*35*)
* pavA*	Adherence and virulence protein A	0/122	0/89	0/33	(*35*)
* fnbA*	Fibronectin-binding protein	0/122	0/89	0/33	(*35*)
Antiphagocytosis					
* cba*	C protein β antigen	0/122	0/89	0/33	(*35*)
Complement protease					
* scpA*	C5a peptidase	122/122 (100)	89/89 (100)	33/33 (100)	(*22*,*35*)
* scpB*	C5a peptidase	122/122 (100)	89/89 (100)	33/33 (100)	(*35*)
Exoenzyme					
* hylB*	Hyaluronidase	122/122 (100)	89/89 (100)	33/33 (100)	(*22*,*35*)
Invasion					
* bca*	C protein α antigen	11/122 (9.0)	5/89 (6)	6/33 (18)	(*35*)
Streptokinases					
* ska*	Streptokinase	122/122 (100)	89/89 (100)	33/33 (100)	(*22*)
* skc*	Streptokinase	122/122 (100)	89/89 (100)	33/33 (100)	(*35*)
* skg*	Streptokinase	122/122 (100)	89/89 (100)	33/33 (100)	(*35*)
Toxins					
* sagA*	Streptolysin S	122/122 (100)	89/89 (100)	33/33 (100)	(*22*)
* slo*	Streptolysin O	122/122 (100)	89/89 (100)	33/33 (100)	(*22*)
* speG*	Streptococcus pyrogenic exotoxin G	81/122 (66.4)	58/89 (65)	23/33 (70)	(*22*)
* cylE*	β hemolysin/cytolysin	0/122	0/89	0/33	(*35*)
* Cfb*	CAMP factor	0/122	0/89	0/33	(*35*)
Other					
* sicG*	Streptococcal inhibitor of a complement	47/122 (38.5)	35/89 (39)	12/33 (36)	(*22*)

### Clinical Outcomes within the Phylogeny

Severe disease features were present in a similar proportion of patients with GCGS disease caused by clade A–E isolates (63%, 40/64 patients) and heterogeneous nonclade isolates (68%, 17/25 patients). There was an observed trend toward increased mortality in patients with isolates from clades A–E (14 deaths) compared with patients with nonclade isolates (4 deaths), although the difference was not statistically significant (p = 0.7698). The number of deaths resulting from GCGS bacteremia caused by the most common clades, A–C (13/55 [24%]), was not significantly different than the number caused by other clades (5/34 [15%]; p = 0.4179). The death rate was also higher among patients with ST15, ST20, and ST17 (26% [14/53 patients]) than among patients with other STs (11% [4/36 patients]), but the difference was not significant (p = 0.1075). 

## Discussion

Our findings from this large study of the genomic epidemiology and molecular determinants of invasive GCGS bacteremia in association with the clinical features and outcomes of disease contribute to an evolving understanding of the changing epidemiology of β-hemolytic streptococcal infections. Similar to the findings of others (*10*), our findings showed that invasive infection is more common among older persons with underlying medical conditions. Although host factors probably contribute to changing epidemiology, enhanced GCGS virulence should be considered a contributor to the rising incidence GCGS bacteremia. We observed rates of severe disease (70%), ICU admission (26%), and toxic shock syndrome (17%) that were higher than those from previous reports, suggesting increased GCGS virulence (*8*). Death occurred among 17 (20%) of the 84 patients with invasive GCGS bacteremia, a finding consistent with those in other reports (*7**–**10*).

As expected, skin and soft tissue infections served as the main portal of entry in more than half the cases of invasive GCGS bacteremia; however, primary bacteremia without alternate sources of infection was seen in a higher proportion (37%) of cases than seen in other reports (*3*,*5*,*14*). Infections without a source of bacteria entry could represent more effective bacterial penetration of skin and mucosal barriers and evasion of the host immune response due to enhanced pathogenic mechanisms.

Organisms in clades B–E were entirely Lancefield group G and had higher rates of invasive infections, possibly suggesting acquired genetic determinants are contributing to increased virulence and evolutionary selection of these clades. However, in this study, no single genetic determinant could account for an organism’s ability to cause invasive infection. Although respiratory tract isolates in our study served as noninvasive controls, they were collected from persons with symptomatic pharyngitis, in whom host defenses might prevent severe infection and invasion into the blood stream. Host defenses may have obscured recognition of a shared invasion factor that could not be detected in our comparisons. 

The virulence factor profiles we described were similar to those previously reported (*11*,*18*,*22*,*23*,*35*). However, *sicG* was present in a substantially higher proportion of isolates in our study (38.5%) than in another study (9.0%) (*18*), and it was primarily within clades C–E. The gene for *bca*, which has only rarely been described in SDSE, was present in a minority of our isolates (9.0%). The superantigen *speG* gene was found to cluster in Lancefield groups C and G, belonging to clades A and B, respectively, and was present in a proportion of isolates similar to that described in other reports (*18*,*22*). The reference isolate, AC-2713, also possessed all 3 of these virulence factors. All other superantigens found in GAS were absent from the isolates in our study.

The toxin gene *sagA* was present in all invasive and noninvasive isolates in our study. Although this toxin has previously been implicated in necrotizing skin and soft tissue infections (*20*), we did not confirm these findings in our study. No cases of necrotizing fasciitis were present in the study cohort; however, skin and soft tissue infections were common and severe, requiring surgical intervention in 17 (19%) of the 89 patients with bacteremia.

A specific cluster within clade C organisms was associated with polymicrobial bacteremia with *S*. *aureus*. All 4 patients co-infected with *S*. *aureus* and clade C GCGS organisms had severe infections: 2 patients, 1 of whom died, required renal replacement therapy; 1 was an intravenous drug user with endocarditis; and 1 was a 60-year-old man with diabetes who sought medical care for STSS from an unknown source and subsequently died. All isolates had *cbp*, *sicG*, and *gfbA* virulence factors. Three of the 4 patients had risk factors for endovascular infection; however, the clustering of these organisms may suggest a synergistic effect of co-infection and invasion with *S*. *aureus*.

Overall, the rising incidence and severity of invasive GCGS infections are probably associated with several evolving bacterial virulence factors. These factors probably take advantage of aging hosts with complex chronic diseases, susceptibilities, and co-existing conditions. Although our findings did not show a single virulence factor to account for emerging virulence, clonal clustering of factors within clades causing invasive infection suggests a survival and invasion advantage over clades without similar virulence clusters. Antimicrobial pressure may lead to accelerated transfer of genetic material, leading to acquisition of virulence factors. Furthermore, it is possible that newly acquired or novel virulence factors not previously described in other β-hemolytic streptococci are present.

In conclusion, the frequency of invasive GCGS infections is surpassing that of GAS infections in patients in Manitoba, Canada, and these infections are associated with severe disease and death. Related strains that cluster clonally are more likely than others to cause invasive disease. The clonal distribution of virulence factors, in combination with host factors, is probably contributing to the emergence of invasive GCGS.

Technical Appendix 1 Genomic methods, single nucleotide variation (SNV) summary of the major phylogenetic clades, details of polymicrobial bloodstream infections, and core SNV phylogenic comparison of all invasive bloodstream and noninvasive respiratory isolates for a study of clonal clusters and virulence factors of group C and G *Streptococcus* causing severe infections, Manitoba, Canada, 2012–2014.

Technical Appendix 2 Dataset representing a matrix of the total single-nucleotide variations between *Streptococcus dysgalactiae* subsp. *equisimilis* isolates from patients with group C and G *Streptococcus* causing severe infections, Manitoba, Canada, 2012–2014.
