# Supplementary material for: Clonal Clusters and Virulence Factors of Group C and G Streptococcus Causing Severe Infections, Manitoba, Canada, 2012–2014
Source: Emerg Infect Dis. 2017 Jul;23(7):1079–88. doi: 10.3201/eid2307.161259 (PMC5512470; doi:10.3201/eid2307.161259)
Supplement: Technical Appendix 1 — Genomic methods, single nucleotide variation (SNV) summary of the major phylogenetic clades, details of polymicrobial bloodstream infections, and core SNV phylogenic comparison of all invasive bloodstream and noninvasive respiratory isolates for a study of clonal clusters and virulence factors of group C and G Streptococcus causing severe infections, Manitoba, Canada, 2012–2014. [file 16-1259-Techapp-s1.pdf]

Article DOI: https://dx.doi.org/10.3201/eid2307.161259

Clonal Clusters and Virulence Factors of Group C and G *Streptococcus* Causing Severe Infections, Manitoba, Canada 2012–2014

Technical Appendix

Methods

Whole Genome Sequencing and Assembly

DNA samples were extracted from cultures following standard protocol with Epicenter Masterpure Complete DNA and RNA Extraction Kit (Mandel Scientific, Guelph, ON). Multiplexed libraries were created with Nextera XT sample preparation kits (Illumina, San Diego, CA). Paired-end, 300 bp indexed reads were generated on the Illumina MiSeq platform (Illumina, San Diego, CA) yielding an average of 1,015,107 reads/genome and average genome coverage of 145X.

De Novo Assembly

The quality of the reads was assessed using FastQC version 0.11.4 (http://www.bioinformatics.babraham.ac.uk/projects/fastqc/), merged using FLASH version 1.2.9 with minimum overlap = 20 and maximum overlap = 300 (*1*), assembled with SPAdes version 3.6.2 (*2*) and annotated with Prokka version 1.11 (*3*). The average contig length generated was 39,313 bp (bp) and the average N50 contig length was 82,867 bp.

Core Single Nucleotide Variation (SNV) Phylogenetic Analysis

FASTQ forward and reverse read files were analyzed using a custom Galaxy Snvphyl paired end fastq workflow (https://github.com/phac-nml/snvphyl-galaxy) with minimum coverage = 15, minimum mean mapping quality = 30, and alternative allele ratio = 0.75. The high-quality reads were then mapped to the publically available reference genome, *Streptococcus dysgalactiae* subsp. *equisimilis* AC-2713 (NCBI Accession NC_019042.1) with SMALT version

0.7.5 (http://www.sanger.ac.uk/resources/software/smalt/) with smalt index K-mer size set to 13 and Step size to 6; and smalt map with maximum insert size = 1000, minimum insert size = 20, and seed = 1. Single nucleotide variants were called using FreeBayes version 0.9.20 (Erik Garrison, Garbor Marth (2012) arXiv:1207.3907[q-bio.GN]) using the following parameters: "–pvar 0–ploidy 1–left-align-indels–min-mapping-quality 30–min-base-quality 30–min-alternate-fraction 0.75–min-coverage 15" with additional variant confirmation using SAMtools mpileup (*4*) and positions where variant calls were not in agreement between both variant callers were excluded. Variant calls within potential problematic regions including repetitive regions identified with Mummer (Galaxy tool version 1.6.1-dev) with minimum length of repeat region set to 150 and minimum PID of repeat region to 90 and highly recombinant regions containing >10 SNVs per 100 bp were removed from the analysis. All remaining variant calls were merged into a single meta-alignment file. The percentage of bases in the core was 82.8% and number of sites used to generate the phylogeny was 21,746.

The meta-alignment of informative core SNV positions was used to create a maximum likelihood phylogenetic tree using PhyML (version 3.0) with generalized time reversible model (*5*) using parameters: Evolution model = "GTR," Branch support = "SH-like aLRT" and Tree topology search operation = "Best of NNI and SPR." The phylogenetic tree was visualized using FigTree version 1.4.1 [http://tree.bio.ed.ac.uk/software/figtree/] and phylogenetic clades were determined by cluster analysis using ClusterPicker version 1.2.4 ***(****6*) with the following settings: initial and main support thresholds = 0.9, genetic distance threshold = 4.5 and the large cluster threshold = 10. Whole-genome sequencing read data was deposited to the NCBI Sequence Read Archive (https://www.ncbi.nlm.nih.gov/sra/) under BioProject accession number PRJNA325743.

References

1. Magoč T, Salzberg SL. FLASH: fast length adjustment of short reads to improve genome assemblies. Bioinformatics. 2011;27:2957–63. http://dx.doi.org/10.1093/bioinformatics/btr507

2. Bankevich A, Nurk S, Antipov D, Gurevich AA, Dvorkin M, Kulikov AS, et al. SPAdes: a new genome assembly algorithm and its applications to single-cell sequencing. J Comput Biol. 2012;19:455–77. http://dx.doi.org/10.1089/cmb.2012.0021

3. Seemann T. Prokka: rapid prokaryotic genome annotation. Bioinformatics. 2014;30:2068–9. http://dx.doi.org/10.1093/bioinformatics/btu153

4. Li H, Handsaker B, Wysoker A, Fennell T, Ruan J, Homer N, et al.; 1000 Genome Project Data Processing Subgroup. The sequence alignment/map format and SAMtools. Bioinformatics. 2009;25:2078–9. http://dx.doi.org/10.1093/bioinformatics/btp352

5. Guindon S, Dufayard J-F, Lefort V, Anisimova M, Hordijk W, Gascuel O. New algorithms and methods to estimate maximum-likelihood phylogenies: assessing the performance of PhyML 3.0. Syst Biol. 2010;59:307–21. http://dx.doi.org/10.1093/sysbio/syq010

6. Ragonnet-Cronin M, Hodcroft E, Hué S, Fearnhill E, Delpech V, Brown AJL, et al.; UK HIV Drug Resistance Database. Automated analysis of phylogenetic clusters. BMC Bioinformatics. 2013;14:317. http://dx.doi.org/10.1186/1471-2105-14-317

Technical Appendix Table 1. Number of single nucleotide variations (SNVs) in the core genome, between and within the major phylogenomic clades of *S. dysgalactiae* subsp. *equisimilis* strains

Clade	No. Isolates	Avg SNPs within clade	Maximum SNPs within clade	Avg SNPs from next clade*	Minimum SNPs from next clade*	Maximum SNPs from next clade*
A	28	95.4	253	1833.6	1671	2031
B	13	291.8	600	341.5	52	619
C	14	67.6	138	1198.9	1159	1292
D	5	104.6	176	3622.9	3578	3677
E	4	290.3	454	3948.8†	3904†	3993†

*Number of SNPs compared to following closest ancestral clade in the phylogeny.
†Number of SNPs compared to clade A in the phylogeny.

Technical Appendix Table 2. Blood isolates of *S. dysgalactiae* subsp. *equisimilis* associated with co-infection with other bacterial organisms in order of clade. Clinical features of disease severity and mortality are included*

Isolate no., clade	Blood culture 1	Blood culture 2	Alternative sources	Severe disease features	Mortality	Potential contaminant
12, No clade	*E. coli,* GGS	*E. coli*	Urine: *E. coli*	Yes	No	Yes
74, No clade	GGS	GGS, CoNS	No	No	No	Yes
27, clade D	*Hafnia alvei, Citrobacter braakii, E. faecium,* GGS	*Hafnia alvei,* GGS	No	No	No	No
85, clade C	*S. aureus* (MRSA), GGS	ND	No	Yes	Yes	No
41, clade C	*S. aureus* (MSSA), GGS	ND	No	Yes	Yes	No
82, clade C	GGS	GGS, *S. aureus* (MSSA)	Wound: 4+ *S. aureus*: 2+ GGS	Yes	No	No
70, clade C	GGS, *S. aureus* (MSSA)	GGS, *S. aureus* (MSSA)	No	Yes	No	No
78, clade B	GGS, GBS	GGS, GBS	Wound: 4+ GGS, 4+ GBS, 1+ CoNS, 1+ *P. multocida*, 3+ *Peptostreptococcus sp.*, 1+ *Propionibacterium sp.*, 1+ *Fusobacterium sp*.	No	No	No
3, clade B	GGS	*Bacillus sp.*	No	Yes	No	Yes
57, clade B	GGS, CoNS	No growth,	No	Yes	No	Yes
60, clade A	*S. aureus* (MRSA), *Bacillus sp.,* GCS	*S. aureus* (MRSA)	Wound: 3+ *S. aureus* (MRSA), 3+ GCS, 1+ *P. aeruginosa*	No	No	No
88, clade A	GCS, *E. coli,* CoNS	ND	No	Yes	Yes	No
42, clade A	GCS, *E. coli*	GCS, *E. coli*	No	Yes	Yes	No
71, clade A	GCS, *S. aureus* (MSSA)	ND	No	Yes	Yes	No
87, clade A	GCS, *P. mirabilis*	ND	No	Yes	Yes	No
38, clade A	GCS, *Lactobacillus paracasei*	*Lactobacillus paracasei, C. albicans*	No	Yes	No	No

*GBS: Group B Streptococcus; GCS: Group C Streptococcus; GGS: Group G Streptococcus; CoNS: Coagulase negative staphylococci; MRSA: Methicillin resistant S. aureus; MSSA: Methicillin sensitive S. aureus; ND: not drawn; Sp: species

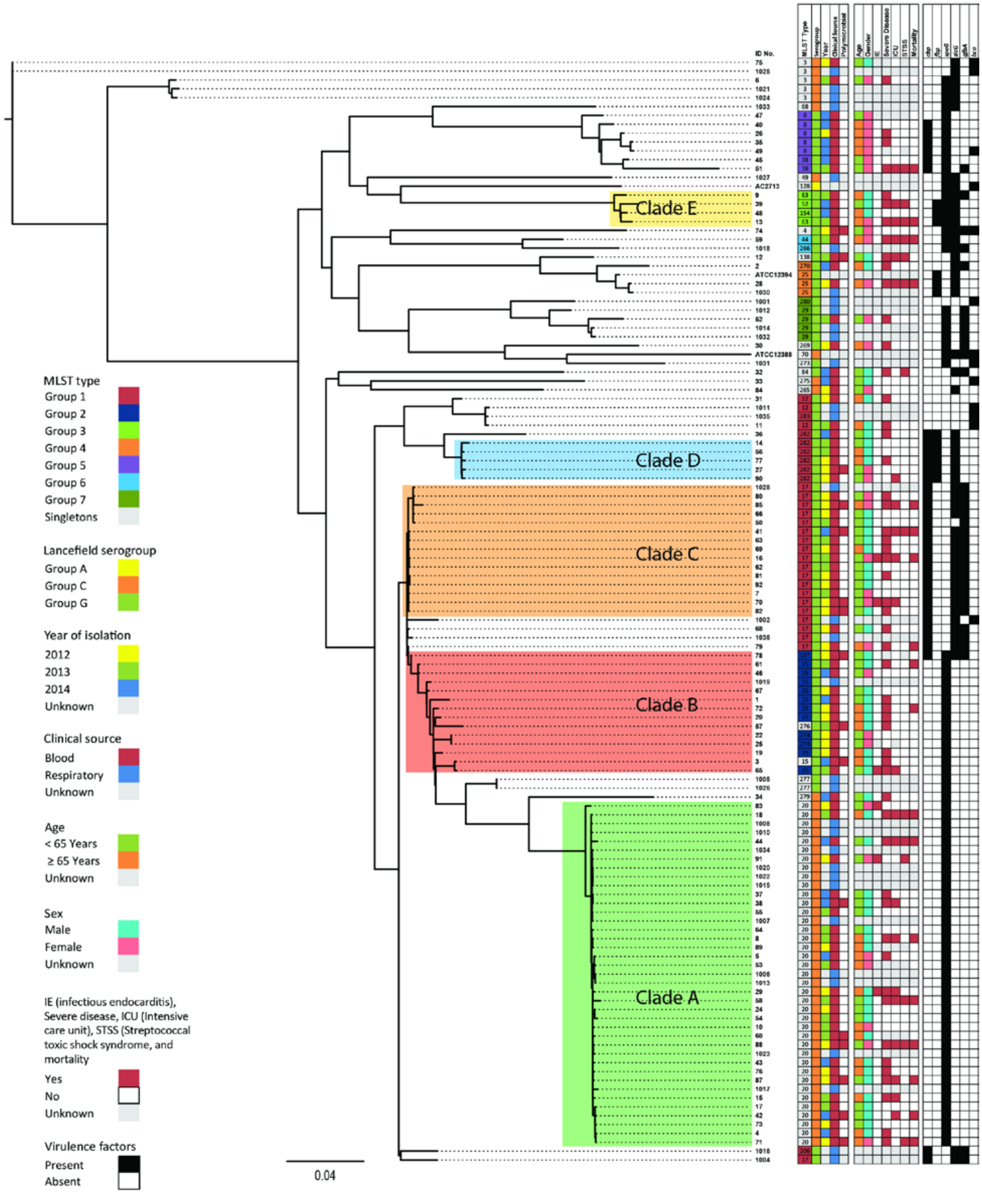

Technical Appendix Figure. Maximum likelihood whole genome core single nucleotide variation (SNV) phylogenetic tree of 89 invasive blood isolates, 33 noninvasive respiratory isolates of *Streptococcus dysgalactiae* subsp. *dysgalactiae* of patients with group C and G streptococci causing severe infections in

Winnipeg, Manitoba, Canada, 2012-2014. Multilocus sequence typing clonal complex relatedness groups were determined by goeBURST (global optimal eBurst; http://www.phyloviz.net/) analysis as presented in Figure 2. Clinical outcome columns indicate severity of disease with red squares indicating high severity, white squares low severity, and gray squares unknown data. Clinical outcome data represented by red squares as yes, white squares as no and grey squares as unknown include: polymicrobial representing coinfection with another organism other than GCGS, IE representing confirmed infectious endocarditis, severe disease represents high risk for in hospital mortality based on the REMS and SCS disease severity scores, ICU represents the need for management in an intensive care unit, STSS as the presence of Streptococcal toxic shock syndrome, and mortality as the death of a patient. Black and white squares for *cbp*, *fbp, speG, sicG gfbA* and *bca* columns represent the presence and absence of virulence factor genes, respectively. The length of the scale bar in the maximum likelihood tree represents the estimated evolutionary divergence between isolates based on the average genetic distance between strains (estimated substitutions in sample/total high quality SNPs).
